# Editorial: Obesity and cancer: update on etiology, molecular biomarkers and biotargets, clinical strategies, and epidemiology

**DOI:** 10.3389/fendo.2023.1258994

**Published:** 2023-08-14

**Authors:** Eva Surmacz, Valentina Guarnotta, Michaela Ruth Reagan

**Affiliations:** ^1^ Allysta Pharmaceuticals, Inc., Belmont, WA, United States; ^2^ Department of Health Promotion, Mother and Child Care, Internal Medicine and Medical Specialties, Section of Endocrinology, University of Palermo, Palermo, Italy; ^3^ Center for Molecular Medicine, MaineHealth Institute of Research (MHIR), Scarborough, ME, United States

**Keywords:** obesity, cancer, animal models, epidemiology—analytic (risk factors), global prevalence

Obesity continues to evolve as a significant global health crisis. It is estimated that by 2025, 2.7 billion adults will be overweight, over 1 billion will be obese, and 177 million will be extremely obese (1, 2). In addition to severe comorbidities like diabetes and cardiovascular disease, the most recent data prove that obesity is associated with developing as many as 18 types of cancer (1, 3). Furthermore, excess body weight increases cancer survivors’ risk of disease recurrence and mortality (4).

Even though human and animal studies validated the link between obesity and cancer, the underlying mechanisms are not entirely understood. At present, vast experimental and evolving clinical evidence links the occurrence of cancer in obese and overweight patients to processes such as chronic inflammation, altered fatty acid metabolism, insulin resistance, abnormal activation of adipokines and anabolic and sex hormones, extracellular matrix remodeling, microbial dysbiosis (1, 4). Nevertheless, new biological pathways are continuously being uncovered and could lead to new perspectives and targets for therapies

In addition to mechanistic considerations, a better understanding of sociological and regional differences in obesity prevalence and cancer risks would be critical in devising the most promising prevention and intervention strategies (5).

This Research Topic includes several reviews and original papers providing updated perspectives on the associations between obesity and cancer. The contributions detail new biomarkers and biotargets, prospective interventions and treatments, and epidemiologic analyses.

Obesity is often associated with metabolic syndrome and diabetes mellitus. Accordingly, several papers in this Research Topic address the link between these pathologies and cancer.

Sun et al. provide new information on the association between prediabetes and diabetes status and breast cancer based on the US National Health and Nutrition Examination Survey. They report that diabetes mellitus is associated with the risk of breast cancer development, and the risk of developing breast cancer increases steadily from non-diabetes to prediabetes and type 2 diabetes. Furthermore, age has a threshold effect on the risk of breast cancer in females, with the risk increasing significantly after age 52.

A systematic review and meta-analysis by Lu and Tao report that diabetes (type 2) and obesity are risk factors for bladder cancer prognosis. The meta-analysis suggests that both diabetes and excessive body weight can negatively influence bladder cancer outcomes such as mortality, progression, and recurrence. However, the risk of mortality due to diabetes in patients with bladder cancer was similar to that in the general population.

Insulin resistance and inflammation have also been shown by Li et al. to be critical mediators of abdominal obesity-related colorectal cancer (CRC) risk. The study reports that C-reactive protein (CRP) and the fasting triglyceride-glucose (TyG) index increased the risk of colorectal cancer independently and synergistically. CRP and the TyG are also reported as mediators for the association between abdominal obesity and CRC risk. These parameters may help clarify the role of abdominal fat disposition over overall obesity in CRC.

A systematic meta-analysis by Zhong et al. suggests a significant relationship between metabolic syndrome and pancreatic cancer. Patients with metabolic syndrome were more likely to develop pancreatic cancer, regardless of gender. The increased risk of developing pancreatic cancer was strongly linked to hypertension, poor high-density lipoprotein cholesterol ratio, and hyperglycemia. However, the prevalence of pancreatic cancer was independent of obesity and hypertriglyceridemia.

This Research Topic also addresses the less well-known relationships between obesity and cancer. For example, the review from Marques-Mourlet et al. examines the clinical and mechanistic impact of obesity on the progression of multiple myeloma (MM). They describe the currently available models for studying obesity in mouse myeloma models and summarize what is known in the field regarding the role of obesity in MM based on epidemiological and preclinical research demonstrating that obesity increases the risk for MM but that the “obesity paradox” persists in terms of outcomes, where obesity does not consistently correlate with worse outcomes.

Chen et al. discuss the links between obesity, non-alcoholic fatty liver disease, and hepatocellular carcinoma. The review focuses on molecular mechanisms and cellular signaling pathways involved in the pathogenesis of obesity-associated hepatocellular carcinoma. The authors also summarize the preclinical, experimental animal models and the non-invasive diagnostic methods of non-alcoholic fatty liver disease and hepatocellular carcinoma and discuss novel therapies for hepatocellular carcinoma in obese patients.

Feng et al. provide novel data on overweight-related transcriptomic signature as a marker for treatment response in hepatocellular carcinoma. Notably, the authors report that the overweight/obesity-associated gene (OAG) signature, including 17 genes, provides reliable performance in the prognosis prediction of hepatocellular carcinoma.

Llanos et al. focus on the molecular mechanisms involved in the association between overall and central body fatness and poorer breast cancer outcomes. The study reports altered gene and/or protein expression of the obesity hormone leptin and its receptor in this process. The authors report that increased body fatness is associated with increased leptin gene expression and elevated leptin receptor levels in breast tumors.

Finally, Zhang et al. review animal models to study obesity and cancer associations. The authors argue that replicating both obesity and malignancy in laboratory animals is extremely difficult. Animals commonly used in obesity research cannot engraft heterolytic tumors. On the other hand, it is challenging to induce obesity in animals commonly used as cancer models. This review summarizes several experimental animal models and protocols that can simultaneously generate obesity and sustain the growth of tumor xenografts.

What is the future of targeting obesity-related cancer? In preventing obesity-associated cancers, weight-reducing strategies such as structured exercise in combination with dietary support and behavior therapy will continue to be the mainstay of interventions. Treatment with glucagon-like peptide-1 analogs and bariatric surgery that produce significant and rapid weight loss might become preventive options in some individuals, such as high-risk patients or selected cancer survivors (1). The discovery of specific obesity-related pathways common for different neoplasms might offer an additional treatment option.

## Author contributions

ES: Conceptualization, Writing – original draft, Writing – review & editing. VG: Writing – original draft, Writing – review & editing. MR: Writing – original draft, Writing – review & editing.
